# Effects of pole position and grip height on upper-body kinetics and throwing performance in paralympic seated shot put

**DOI:** 10.3389/fspor.2026.1859378

**Published:** 2026-06-23

**Authors:** Rony Ibrahim, Connor J. M. Holdback, David S. Haydon, Richard M. Kelso, Ross A. Pinder, Paul N. Grimshaw

**Affiliations:** 1 College of Sport Sciences, Qatar University, Doha, Qatar; 2 School of Electrical and Mechanical Engineering, Adelaide University, Adelaide, SA, Australia; 3 South Australian Sports Institute, Mile End, SA, Australia; 4 Australian Athletics, Albert Park, VA, Australia; 5 Performance Insights & Innovation, Paralympics Australia, Adelaide, SA, Australia; 6 AIS Performance, Australian Institute of Sport, Canberra, ACT, Australia; 7 College of Health and Life Sciences, Hamad Bin Khalifa University, Doha, Qatar; 8 School of Allied Health and Human Performance, College of Health, Adelaide University, Adelaide, SA, Australia

**Keywords:** biomechanics, equipment optimisation, inverse dynamics, motor redundancy, paralympic sport, pole force, seated throwing

## Abstract

**Purpose:**

This study investigated the effects of pole position (horizontal distance from the seat) and pole grip height on kinetic parameters and throwing performance in Paralympic seated shot put.

**Methods:**

Four F33–34 athletes (1 national and 3 international levels) each performed 27 maximum-effort throws across nine pole configurations (3 pole positions × 3 grip heights) in a fully crossed within-subject design. Upper-body joint powers and pole forces were calculated using three-dimensional inverse dynamics and an instrumented throwing pole. Linear mixed models with random intercepts for athlete were used to assess the effects of pole position and grip height on 13 kinetic variables, with Benjamini–Hochberg false discovery rate correction. Within-athlete performance associations were tested for variables with significant pole configuration effects. A post-protocol fatigue check (three additional throws at each athlete's normal configuration) confirmed no significant performance decrement (d = 0.24).

**Results:**

Pole position significantly affected seven of thirteen kinetic variables (all q < 0.05), including trunk axial rotation power (d = 1.15), throwing-arm shoulder power, and pole forces (posterior pole force d = 2.67). No grip height main effects or interactions survived correction. Despite these substantial kinetic changes, throw distance was not significantly affected by any pole configuration (marginal R^2^ = 0.005). Of the seven significant variables, only mean lateral pole force was associated with within-athlete throw distance (*β* = −0.043 m/N, q = 0.005), with a more laterally directed force linked to greater distance.

**Conclusion:**

Pole position is the primary equipment variable influencing kinetic strategy in seated shot put, while athletes maintain comparable throw distances through compensatory movement strategies. Mean lateral pole force is the sole kinetic variable that is both sensitive to pole position and predictive of performance, providing preliminary evidence that lateral pole force may be a useful candidate variable for individualised pole position selection, although the practical magnitude (within-athlete *Δ*R^2^ = 0.012) is modest. Pole configuration can be leveraged as a targeted training tool to modulate joint loading without compromising competitive performance.

## 1 Introduction

Kinetic analysis of seated shot put has been enabled through the methods outlined in recent research using an instrumented throwing pole (1). Previous findings show that varying the pole grip height can increase trunk power and thus the potential for improved performance (2), adding to earlier research that highlighted that pole position (horizontal distance from the seat) can impact trunk kinematics in seated shot put (3). These results were based on measured outcomes from single athlete case-studies, and it is uncertain whether such findings are consistent across multiple athletes within the F33–34 classification. A logical next step is to identify how pole position and grip height can impact performance across multiple athletes to provide preliminary multi-athlete evidence relevant to athlete-specific equipment optimisation within the F33–34 classification. In addition, the research so far has measured the impact of changing pole position or changing pole grip height, but not a combination of these options (2, 3). Therefore, this study will measure the kinetics of multiple athletes while using nine different athlete-pole configurations: three pole positions (close, normal, far) and three grip heights (low, normal, high).

More broadly, Paralympic throwing biomechanics rests on a small but informative supporting literature. Early work on the seated throwing frame demonstrated that frame configuration meaningfully shapes performance in elite male seated shot-putters (4), and medical classification has been shown to account for substantial between-athlete variability even in mechanically simpler events such as wheelchair discus (5). In wheelchair sports more generally, upper-extremity force production is governed by the interplay between the athlete's residual function and the surface they interact with, an interaction that has received sustained attention in propulsion-based events but comparatively little in throwing (6). Equipment-optimisation work in the wheelchair court sports has further emphasised that small adjustments to seat-to-implement geometry can substantially alter force-generating posture, and that optimal configurations differ between athletes within a class (7). These findings are consistent with the evidence-based principles of paralympic classification, which require demonstrable links between impairment, technique, and performance-determining mechanics (8). Against this background, seated shot put is unusual in that the athlete-pole interface is a load-transmitting fixture rather than a propulsion contact, and its mechanical effects on the throw remain comparatively under-characterised.

Comparing the performance of athletes in different positions with different conditions is difficult unless there are multiple tests over time for each athlete. The movement pattern in seated shot put is a complex culmination of joint and segment motion that requires practice when any change is implemented. O’Riordan et al. (3) demonstrated this whereby changing the pole placement increased trunk rotational velocity but not overall performance of an athlete. Similarly, Holdback et al. (2) demonstrated this in a case study, showing that changing the grip height did not produce an immediate performance improvement, but an improvement was realised after several sessions. Once the athlete had time to practice with the new grip height, they showed a trend towards improved performance. This highlighted the value in measuring athlete biomechanical parameters to seek advantageous pole adjustments, tracking performance over longer periods of time and not simply relying on single testing sessions. We acknowledge that the present study is itself a single-session protocol; the findings reported here should therefore be interpreted as acute biomechanical responses to configuration change rather than as evidence about chronic adaptation or learned configurations.

Athlete impairment is very specific to the individual, and this can have a profound impact on how they perform the movements in their sport. Classification in Paralympic sport is used to group athletes based on impairment type and severity with the goal to ensure fair competition (9). Seated throwing classifications are organised into two distinct groups, with athletes assigned to discrete classes within each group based on the type and severity of their impairment. One group (classes F31–34) includes athletes that have coordination impairments, while the other group (classes F51–57) includes athletes that have impairments such as muscle weakness or limb difference (inter-limb length difference or amputee). Each of these different impairment types presents unique functional limitations. For example, classes F51-F57 include athletes with spinal cord injuries who may have significant trunk weakness especially in the lower classes (F51–53), while classes F31–34 often have less significant trunk weakness, and this is an important distinction that can influence throwing technique and equipment (10). Variation between athletes is likely to impact the ability for research to produce findings that are generalisable across different classes and especially impairment types. Therefore, it may be reasonable to conduct analysis on a group of athletes with similar impairment types before investigating cohorts with a mix of different impairments. Overall, it is likely that athletes within similar classifications will have large amounts of between-athletes variability when compared to non-disabled athletes and this should be considered when conducting research in Para-sport.

The present study aims to investigate how pole position and grip height impact the kinetic parameters (joint power) and performance (throw distance) of athletes in seated shot put. The approach will not be constrained to only trunk parameters, as done previously (2), but instead will consider the trunk, throwing-arm shoulder/elbow, pole-arm shoulder/elbow, and three-component pole forces. Since past research has shown pole position to have a similar effect on trunk parameters (2, 3), it was hypothesised that: (1) pole position would, at the group level, significantly affect upper-body kinetic parameters; (2) grip height would also influence kinetic parameters, though potentially to a lesser extent; and (3) the kinetic changes induced by pole configuration would be associated with changes in throwing performance.

## 2 Materials and methods

### 2.1 Study design and data collection

Four athletes (Class F33–34; 2-Male, 2-Female; 1 national-level, and 3 international-level competitors) were recruited in collaboration with their National Sporting Organisation. Further specifics regarding individual athletes are not disclosed in this manuscript to prevent re-identification within a sport with a small competitive pool. The study was approved by University of Adelaide Human Ethics application (H-2022-147), and it was conducted in accordance with the local legislation and institutional requirements. Athletes provided their written informed consent to participate in this study. All athletes reported no injuries or recent changes to their equipment. Each athlete performed maximum-effort throws using nine different pole positions: 150 mm closer than their normal position, their normal position, and 150 mm further than their normal position. For each of these positions, there were three variations of grip height: the athletes’ normal grip height, a grip 75 mm lower than their own normal, and a grip 75 mm higher than their own normal. The magnitudes of the changes in pole placement and grip height were chosen in conjunction with the seated-throwing coaches to be large enough to see notable changes in throwing technique, while still being comfortable and practical. The 150 mm pole-position increment was selected to produce a clearly perceivable change in the horizontal moment arm while remaining within the range previously reported as athlete-tolerable in a single-session protocol (2); the smaller 75 mm grip-height increment reflected the narrower range of grip variation that coaches identified as ergonomically feasible without re-fitting the pole assembly. Three throws for each grip height were performed in a random order for each pole position (close, normal, far). The order of the three pole-position blocks was constrained by the requirements of a single-session protocol rather than fully counterbalanced. The Normal block was tested first for every athlete, both to establish the reference frame position from which the ±150 mm Close and Far shifts were subsequently measured and to provide opening Normal-condition throws for the end-of-session fatigue comparison. The Close and Far blocks were then alternated between athletes, and the session closed with a return-to-Normal block used as the within-protocol fatigue check. While complete randomisation of order would be ideal, it is important to understand the set-up required for athletes in seated throwing; athletes need to transfer onto the throwing frame from either standing or from their wheelchair, after which strapping is applied around their legs and hips. This process is typically only performed once in competition before they perform their six throws. Ideally for a fully-counterbalanced experimental design, the order of testing positions would be completely randomised, with the pole position and grip height varying with each throw. However, due to the need for the athlete to dismount from the frame each time, this was adjusted such that the pole position was fixed in each of the close, normal, or far positions for nine throws at a time (the three throws for each of the three grip heights). In total, this resulted in 27 throws for each athlete, where the throwing distances were recorded for each throw. The athletes were asked to rest as long as necessary to ensure they could perform maximally on the next throw. In addition, to assess any significant impact from fatigue, a further 3 throws were added to the end of the protocol (performed with each athlete's normal pole position and grip height) to quantify any performance degradation over the testing period.

Testing was performed outdoors in an undercover area mitigating the effects of wind and weather. Athletes used their own throwing frame with the only modification being that their pole was removed and an instrumented pole was placed in the same position relative to their frame. This allowed the athlete to use their normal strapping and foot supports. The instrumented pole incorporated a ME Systems K3D120 load cell (1,000 Hz) for measuring the three-component pole forces during the throw: medio-lateral, antero-posterior and vertical (1). Each athlete used the World Para Athletics regulation shot mass specified for their classification and sex (9).

### 2.2 Modelling and kinetics

Upper-body kinematics (elbow, shoulder, and trunk) were recorded using a Vicon motion capture system with twelve V8 Vantage cameras (200 Hz) and 23 retroreflective markers positioned on the body (Appendix A). Following this, segment coordinate systems were defined according to recommendations from Cappozzo et al. (11) with joint rotations defined in the local coordinate system of the proximal segment (Appendix B and C). The athlete was then modelled as a ‘linked segment model’ (12) with the trunk as the proximal segment and the throwing hand as the distal segment (wrist joint centre as the distal endpoint). A dynamic three-dimensional (3-D) linked segment model was used to calculate via top-down inverse dynamics techniques Elbow, Shoulder, and Trunk 3-D power. Details of the original model and the modifications for more accurate anatomical modelling of the body segments can be found elsewhere (13–16). In total, 13 kinetic variables were evaluated: average lateral pole force, average posterior pole force, peak trunk power (3D), peak shoulder power (3D; throwing-arm and pole-arm), peak elbow power (1D: flexion-extension; throwing-arm and pole-arm). Synchronisation of the kinematic (200 Hz) and force (1000 Hz) data streams, resampling, and the low-pass filtering followed the pipeline described and validated in Holdback et al. (1); the present study used this pipeline without modification. Specifically, marker trajectories were low-pass filtered with a zero-phase fourth-order Butterworth filter prior to differentiation, with the cut-off frequency selected by residual analysis as in Holdback et al. (1); the same reference describes the synchronisation hardware and the resampling approach in full numeric detail. Movement onset was identified as the power position, indicated by a peak in the measured pole total force (2), and the end of the throw was defined as the point of indicated by the peak throwing-hand wrist joint centre linear velocity. The ‘mean’ pole-force variables (mean lateral and mean posterior pole force) were calculated as the arithmetic mean of the corresponding force component across the propulsive phase from movement onset to release; peak power values were extracted as the maximum instantaneous joint power within the same window. These event-detection and phase-windowing conventions follow those used in Holdback et al. (2).

### 2.3 Statistical analysis

All statistical analyses were performed in R (version 4.5; R Core Team, 2025) using the *lme4*, *lmerTest*, *emmeans*, *MuMIn*, *effectsize*, *car*, and *performance* packages. Significance was set at *p* < 0.05 throughout, with corrections for multiple comparisons as described below.

#### 2.3.1 Fatigue

To evaluate whether fatigue influenced throwing performance across the testing session, three additional throws were performed using the normal pole position and normal grip height immediately following the experimental protocol. These post-protocol throws were compared with the during-protocol throws performed under the same configuration using a Linear Mixed-Model (LMM) with timepoint (during vs. post) as a fixed effect and athlete as a random intercept. Cohen's d and 95% confidence intervals for the mean difference were reported.

#### 2.3.2 Pole configuration

The effects of pole position (Close, Normal, Far) and grip height (Low, Normal, High) on each of the 13 kinetic dependent variables were examined using separate linear mixed models (LMMs). Each model included pole position, grip height, and their interaction as fixed effects, with a random intercept for athlete to account for repeated measures and between-athlete variability in baseline kinetic profiles. Models were fitted using restricted maximum likelihood (REML) estimation, and degrees of freedom were approximated using the Satterthwaite method for Type III *F*-tests. A random-intercept-only structure was employed because the small number of athletes (*n* = 4) precluded the estimation of random slopes without introducing convergence failures or singular fits. All analyses were conducted on raw (unnormalised) data, as the random intercept adequately partitions between-athlete variance. An identical LMM was also fitted for throw distance to assess whether pole configuration directly affected performance.

#### 2.3.3 Power, sensitivity, and reliability

A *post-hoc* sensitivity analysis indicated that, with 99 observations across four athletes and 87 residual degrees of freedom, the present design has approximately 80% power to detect Cohen's f ≈ 0.32 (*η*^2^ ≈ 0.09) at uncorrected *α* = 0.05, increasing to Cohen's f ≈ 0.39 (*η*^2^ ≈ 0.13) at the more stringent threshold corresponding to FDR-adjusted q < 0.05. For pairwise contrasts within a 3-level factor under a balanced design this corresponds to a minimum reliably detectable Cohen's d of approximately 0.78–0.94; effects below this would not reliably reach significance. Within-condition, within-athlete reliability was characterised for the principal outcome variables by computing the typical error (TE), pooled as the square root of the mean within-cell variance, and the median coefficient of variation (CV%) across the 36 athlete-by-condition cells. Throw distance (TE = 0.31 m, CV = 2.6%), mean lateral pole force (TE = 1.21 N, CV = 5.9%) and mean posterior pole force (TE = 2.36 N, CV = 5.4%) exhibited acceptable trial-to-trial reliability, whereas trunk and shoulder powers showed moderate to high trial-to-trial variability (median CV 11%–27%). With only 2–3 throws per cell these reliability estimates carry wide uncertainty, and we did not compute ICC(3,1) values because the degree-of-freedom requirements are not met under such limited replication.

#### 2.3.4 Multiple comparison corrections

Each model yielded three *p*-values (pole position main effect, grip height main effect, and their interaction). To control the family-wise false discovery rate across the 13 kinetic variables, Benjamini-Hochberg (BH) false discovery rate (FDR) correction was applied separately within each effect type (i.e., 13 *p*-values for pole position, 13 for grip height, and 13 for the interaction), yielding FDR-corrected q-values. Effects were considered significant at q < 0.05.

#### 2.3.5 *Post-Hoc* comparisons and effect sizes

For each kinetic variable with at least one significant fixed effect, *post-hoc* pairwise comparisons were conducted on estimated marginal means (EMMs) using the *emmeans* package with Tukey adjustment for three-level comparisons. For significant interactions, simple effects were examined (i.e., the effect of pole position at each grip height level, and vice versa). Cohen's d effect sizes and their 95% confidence intervals were computed for all pairwise contrasts using the residual standard deviation from the LMM as the standardiser. Marginal R^2^ (variance explained by fixed effects) and conditional R^2^ (variance explained by fixed and random effects combined) were extracted from each model using the *MuMIn* package. Intraclass correlation coefficients (ICCs) were computed as the ratio of athlete-level variance to total variance to quantify the proportion of variability attributable to between-athlete differences.

#### 2.3.6 Performance association

To assess whether kinetic variables that were significantly influenced by pole configuration also predicted throwing performance, within-athlete associations were tested for each significant variable. Each kinetic variable was centred within each athlete (by subtracting the athlete's mean across all conditions), and the centred variable was entered as a fixed-effect predictor of throw distance in an LMM with a random intercept for athlete. This within-athlete centring isolates the within-athlete relationship between kinetic changes and performance changes, removing confounding by between-athlete differences. The change in marginal R^2^ relative to a null model (intercept and random effect only) was reported as a measure of the explained within-athlete variance. FDR correction (Benjamini-Hochberg) was applied across the set of surviving kinetic variables.

#### 2.3.7 Model diagnostics and sensitivity analyses

Model assumptions were evaluated through inspection of quantile-quantile (QQ) plots and residual-versus-fitted-value plots. Residual normality was formally tested using Shapiro–Wilk tests, and homoscedasticity was assessed using Levene's test on model residuals grouped by experimental condition. Singular fits (boundary estimates of random-effect variances) were checked for all models. Influential observations were identified using Cook's distance, with 4/n as the threshold.

Two sensitivity analyses were conducted to evaluate the robustness of findings to violations of normality. First, for all models with non-normal residuals (Shapiro–Wilk *p* < 0.05), the dependent variable was log-transformed (with an additive offset where necessary to handle non-positive values) and the LMM was re-fitted; conclusions (significant vs. non-significant at *p* < 0.05) were compared between original and log-transformed models. Second, a within-athlete permutation test (1,000 permutations) was performed for each significant effect: on each iteration, the dependent variable values were randomly shuffled within each athlete (preserving the random-effect structure), and the F-statistic was re-computed. The permutation *p*-value was calculated as the proportion of permuted F-statistics exceeding the observed value.

## 3 Results

A total of 99 valid throws were analysed across 4 athletes, 3 pole positions, and 3 grip heights. A throw was retained as valid if (i) all required upper-body marker trajectories were continuous (with no occlusion), (ii) the pole-force trace was continuous from athlete contact to release, and (iii) the shot landed within the official throwing sector. Throws with marker occlusions, incomplete pole-force traces, or sector faults were excluded prior to analysis. Cell sizes ranged from 2 to 3 throws per athlete per condition. Mean throw distance was similar across all nine conditions, ranging from 6.32 ± 0.98 m (Far/Normal) to 6.51 ± 1.15 m (Close/Normal) (Table 1). The LMM for throw distance did not detect a significant effect of pole position [F(2, 87.0) = 2.24, *p* = 0.113], grip height [F(2, 87.0) = 0.02, *p* = 0.982], nor their interaction [F(4, 87.0) = 0.23, *p* = 0.922] on throw distance. The high conditional R^2^ and ICC (0.92) indicated that the vast majority of variance in throw distance was attributable to between-athlete differences rather than experimental conditions. In absolute terms, the fixed effects of pole configuration explained approximately 0.5% (marginal R^2^ = 0.005) of the throw-distance variance, with the remainder attributable to between-athlete differences (ICC=0.92). The analysis of configuration effects in this dataset is therefore strongly dominated by individual differences in baseline performance, and configuration-level comparisons should be interpreted on a within-athlete basis.

**Table 1:** Mean and standard deviation (SD) for throw distance by condition.

Pole Position	Grip Height	Mean	SD	*n*
Close	Low	6.49	1.25	12
Close	Normal	6.51	1.15	12
Close	High	6.47	1.11	8
Normal	Low	6.42	0.97	10
Normal	Normal	6.42	0.81	11
Normal	High	6.38	0.78	11
Far	Low	6.41	0.88	12
Far	Normal	6.32	0.98	12
Far	High	6.38	1.03	11

**Figure 1:**
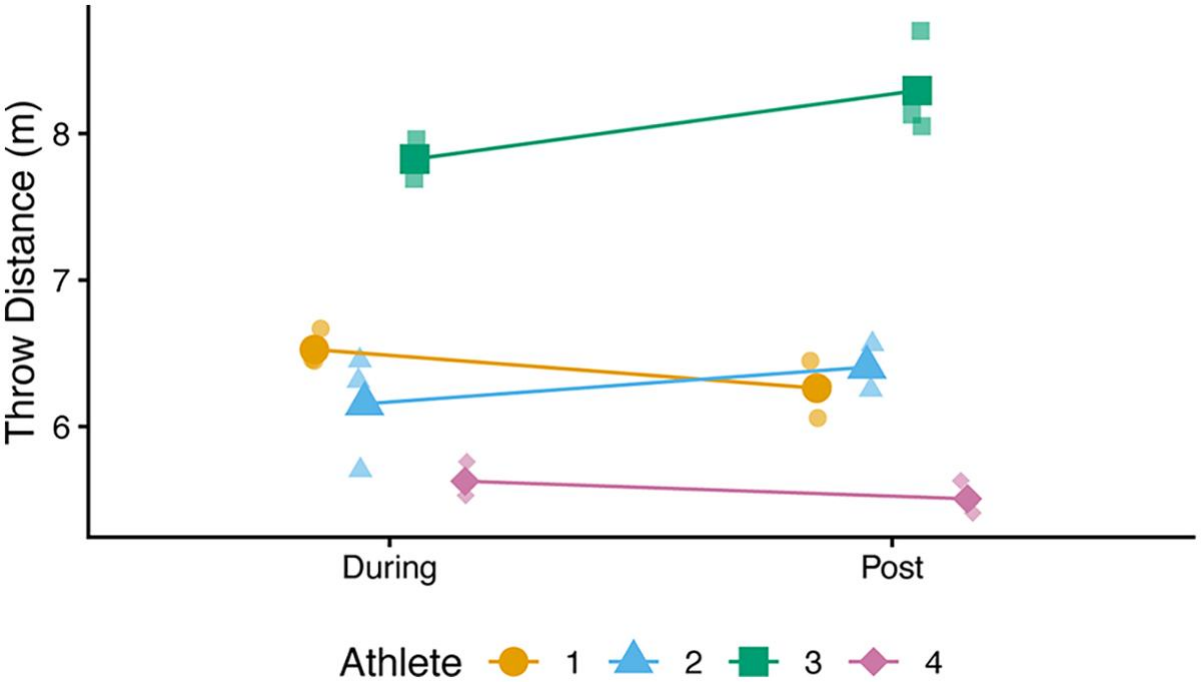
Fatigue evaluation: throw distance during- vs. post-protocol. Large solid colour markers = condition means, small faded colour markers = individual trials.

To assess potential fatigue effects, throw distance was compared between during-protocol (*n* = 11 throws) and post-protocol (*n* = 12 throws) trials using an LMM with timepoint as a fixed effect and athlete as a random intercept (Figure 1). No significant difference was observed [mean difference=0.07 m, 95% CI ( −0.17, 0.30), t (18.0) = 0.58, *p* = 0.566, d = 0.24], indicating that fatigue did not systematically affect throwing performance across the testing session.

### 3.1 Effect of pole configuration on kinetic variables

**Figure 2:**
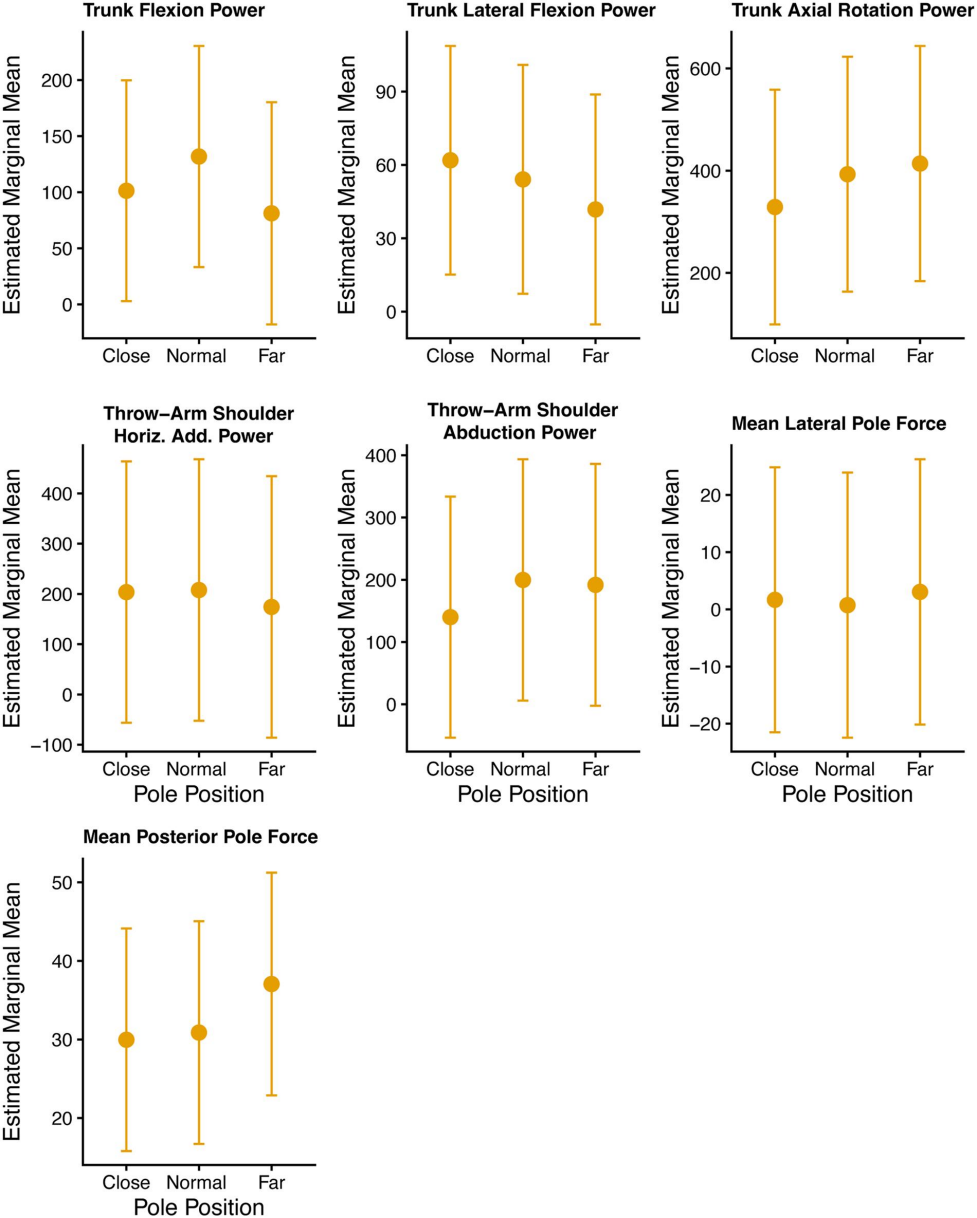
Estimated marginal means for significant kinetic variables. Error bars represent 95% confidence intervals.

**Figure 3:**
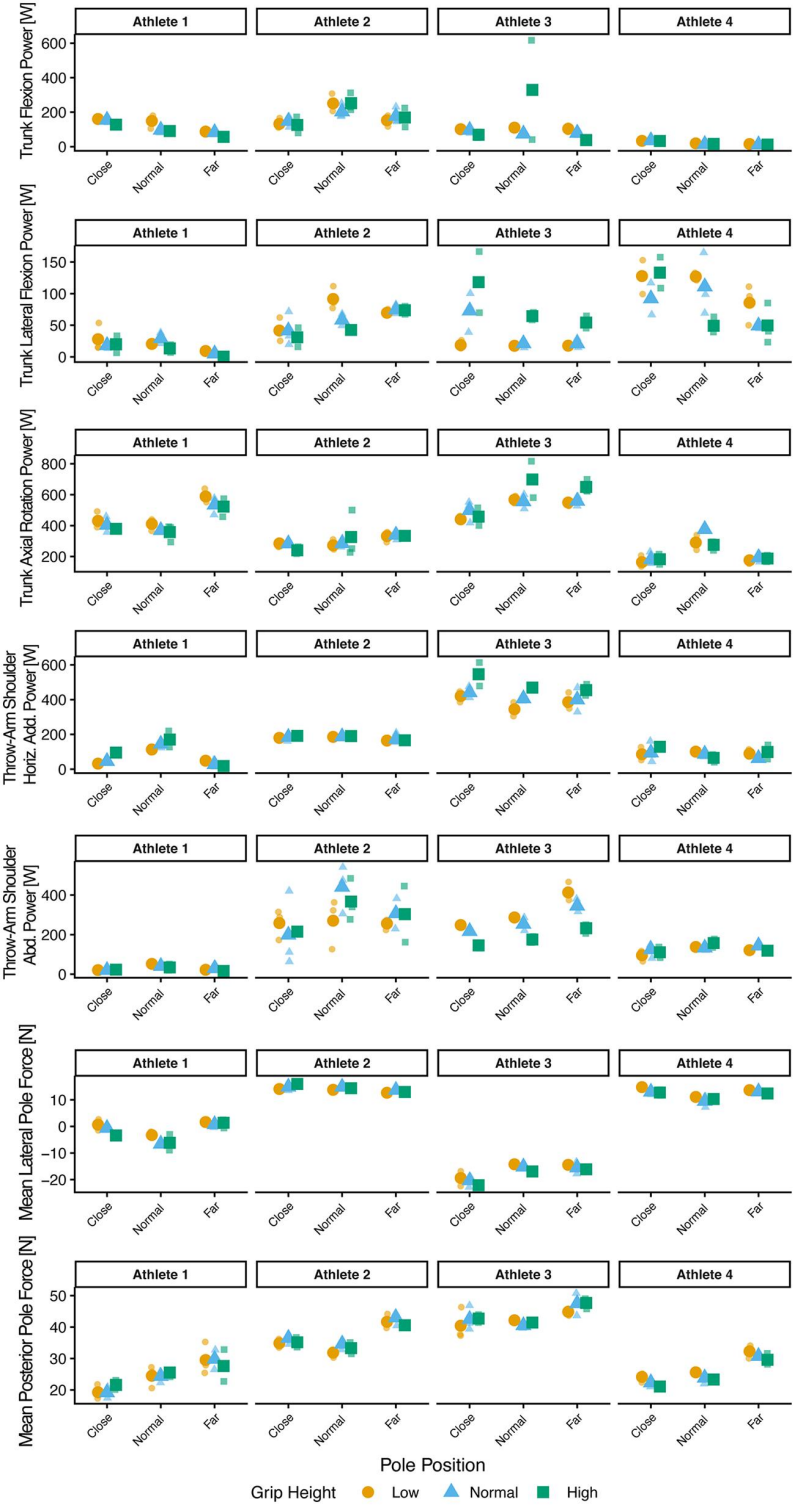
Individual athlete patterns. Large solid colour markers = condition means, small faded colour markers = individual trials.

LMM results for all 13 kinetic variables are presented in Table 2, Figure 2 for group results and Figure 3 for within-athlete results. Seven variables exhibited a significant main effect of pole position after FDR correction (q < 0.05): trunk flexion power, trunk lateral flexion power, trunk axial rotation power, throwing-arm shoulder horizontal adduction power, throwing-arm shoulder abduction power, mean lateral pole force, and mean posterior pole force. No significant main effects of grip height or pole position  ×  grip height interactions were observed for any variable after FDR correction (all q > 0.05). ICCs ranged from 0.31 (pole-arm elbow flexion power) to 0.98 (mean lateral pole force), indicating substantial between-athlete variability across all kinetic measures.

**Table 2:** Effect of pole position (PP) and grip height (GH) on kinetic variables (13 linear mixed models).

Variable	PP F(2, 87)	PP q	GH F(2, 87)	GH q	Inter F(4, 87)	Inter q	Marginal R2	Conditional R2	ICC athlete
Trunk Flexion Power*	5.36	**0**.**0138**	0.37	0.8668	1.41	0.9382	0.079	0.5399	0.5004
Trunk Lateral Flexion Power*	3.98	**0**.**0411**	0.22	0.8668	1.83	0.9382	0.0678	0.5468	0.5139
Trunk Axial Rotation Power*	11.52	**0**.**0002**	0.14	0.8668	0.35	0.9382	0.0484	0.8043	0.7944
Pole-Arm Shoulder Horiz. Add. Power	0.02	0.9842	0.3	0.8668	0.2	0.9382	0.0067	0.5635	0.5606
Pole-Arm Shoulder Extension Power	1.22	0.3918	3.25	0.1892	0.2	0.9382	0.0586	0.4545	0.4205
Pole-Arm Shoulder Adduction Power	0.25	0.8466	2	0.3086	0.95	0.9382	0.0507	0.4608	0.432
Pole-Arm Elbow Flexion Power	2	0.2301	3.87	0.1598	0.69	0.9382	0.0872	0.3735	0.3136
Throw-Arm Shoulder Horiz. Add. Power*	6.42	**0**.**0065**	5.86	0.0534	1.07	0.9382	0.0167	0.9395	0.9384
Throw-Arm Shoulder Flexion Power	0.29	0.8466	1.99	0.3086	0.36	0.9382	0.0093	0.8565	0.8552
Throw-Arm Shoulder Abduction Power*	6.58	**0**.**0065**	1.37	0.4759	0.51	0.9382	0.0431	0.7606	0.7498
Throw-Arm Elbow Flexion Power	1.81	0.2464	1.25	0.4759	1.06	0.9382	0.0512	0.4491	0.4194
Mean Lateral Pole Force*	8.57	**0**.**0017**	2.39	0.3086	0.26	0.9382	0.0057	0.9759	0.9757
Mean Posterior Pole Force*	70.8	**0**	0.29	0.8668	0.28	0.9382	0.1083	0.9276	0.9188

The bold values correspond to *q* < 0.05.

* Added to the variable when *q* < 0.05.

#### 3.1.1 Trunk kinetic variables

Pole position significantly affected trunk axial rotation power [F(2, 87.0) = 11.52, *p* < 0.001, q < 0.001, marginal R^2^ = 0.048, conditional R^2^ = 0.804]. *post-hoc* comparisons revealed that trunk axial rotation power was significantly greater in the Far condition (estimated marginal mean, EMM=414 W) than the Close condition [EMM=329 W; difference=85.0 W, d = 1.15, 95% CI (0.63, 1.67), *p* < 0.001], and significantly greater in the Normal condition (EMM=393 W) than the Close condition [difference=64.2 W, d = 0.87, 95% CI (0.35, 1.39), *p* = 0.003]. The Far and Normal conditions did not differ significantly (*p* = 0.487).

Trunk flexion power was also significantly affected by pole position [F(2, 87.0) = 5.36, *p* = 0.006, q = 0.014, marginal R^2^ = 0.079, conditional R^2^ = 0.540]. The Normal condition (EMM = 131.8 W) produced significantly greater trunk flexion power than the Far condition [EMM = 81.2 W; difference = 50.7 W, d = 0.80, 95% CI (0.30, 1.30), *p* = 0.005]. No other pairwise comparisons reached significance.

Trunk lateral flexion power showed a significant pole position effect [F(2, 87.0) = 3.98, *p* = 0.022, q = 0.041, marginal R^2^ = 0.068, conditional R^2^ = 0.547]. This was driven by significantly greater trunk lateral flexion power in the Close condition (EMM = 61.9 W) than the Far condition [EMM = 41.8 W; difference = 20.1 W, d = 0.69, 95% CI (0.19, 1.19), *p* = 0.018].

#### 3.1.2 Throwing-arm kinetic variables

Throwing-arm shoulder horizontal adduction power was significantly influenced by pole position [F (2, 87.0) = 6.42, *p* = 0.002, q = 0.006, marginal R^2^ = 0.017, conditional R^2^ = 0.94]. The high conditional R^2^ reflected substantial between-athlete variability (ICC = 0.94). *post-hoc* comparisons showed that horizontal adduction power was significantly greater in the Normal (EMM = 208 W) and Close (EMM = 204 W) conditions compared with the Far condition [EMM = 174 W; Normal vs. Far: difference = 33.7 W, d = 0.80, 95% CI (0.30, 1.30), *p* = 0.004; Close vs. Far: difference = 29.5 W, d = 0.70, 95% CI (0.20, 1.21), *p* = 0.015]. Grip height approached but did not reach FDR-corrected significance [F(2, 87.0) = 5.86, *p* = 0.004, q = 0.053].

Throwing-arm shoulder abduction power also showed a significant pole position effect [F(2, 87.0) = 6.58, *p* = 0.002, q = 0.006, marginal R^2^ = 0.043, conditional R^2^ = 0.761]. Normal (EMM = 200 W) and Far (EMM = 192 W) conditions elicited significantly greater abduction power than the Close condition [EMM = 140 W; Normal vs. Close: difference = 59.8 W, d = 0.84, 95% CI (0.32, 1.36), *p* = 0.004; Far vs. Close: difference = 51.9 W, d = 0.73, 95% CI (0.23, 1.23), *p* = 0.011].

#### 3.1.3 Pole forces

Mean posterior pole force showed the strongest pole position effect of all variables [F (2, 87.0) = 70.80, *p* < 0.001, q < 0.001, marginal R^2^ = 0.108, conditional R^2^ = 0.928]. The Far condition (EMM = 37.1 N) produced substantially greater posterior pole force than both the Close [EMM = 30.0 N; difference = 7.1 N, d = 2.67, 95% CI (2.04, 3.31), *p* < 0.001] and Normal conditions [EMM = 30.9 N; difference = 6.2 N, d = 2.33, 95% CI (1.73, 2.93), *p* < 0.001]. These were the largest effect sizes observed in the study.

Mean lateral pole force was also significantly affected by pole position [F(2, 87.0) = 8.57, *p* < 0.001, q = 0.002, marginal R^2^ = 0.006, conditional R^2^ = 0.976]. The Far condition (EMM = 3.05 N) differed significantly from the Normal condition [EMM = 0.74 N; difference = 2.32 N, d = 1.01, 95% CI (0.50, 1.52), *p* < 0.001] and the Close condition [EMM = 1.67 N; difference = 1.38 N, d = 0.60, 95% CI (0.10, 1.10), *p* = 0.044]. The very high ICC (0.98) for this variable indicated that lateral pole force magnitudes and directions were highly athlete-specific, reflecting individual technique differences.

### 3.2 Performance association

**Figure 4:**
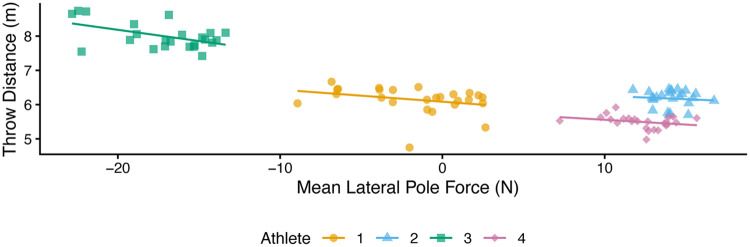
Within-Athlete performance associations. Lines show within-athlete linear trends.

Of the seven kinetic variables with significant pole position effects, only mean lateral pole force showed a significant within-athlete association with throw distance after FDR correction [beta = −0.043, 95% CI (−0.068, −0.019), t(94.0) = −3.53, *p* < 0.001, q = 0.005, *Δ*R^2^ = 0.012; Table 3, Figure 4]. The negative coefficient indicates that a within-athlete shift toward more negative (i.e., more laterally directed) pole force was associated with greater throw distance, with each 1 N decrease in mean lateral pole force corresponding to an increase of 4.3 cm in throw distance. No other kinetic variable showed a significant within-athlete performance association (all q > 0.35; Table 3). The *Δ*R^2^ of 0.012 indicates that within-athlete shifts in mean lateral pole force explain approximately 1.2% of within-athlete throw distance variance, a statistically robust but practically modest association.

**Table 3:** Performance association for significant variables from the effect of pole configuration.

Variable	95% CI	Delta R2m	p FDR
Trunk Flexion Power	[−0.001, 0.001]	0	0.8449
Trunk Lateral Flexion Power	[−0.001, 0.004]	0.002	0.3659
Trunk Axial Rotation Power	[−0.001, 0]	0.001	0.4706
Throw-Arm Shoulder Horiz. Add. Power	[0, 0.002]	0.0022	0.3659
Throw-Arm Shoulder Abduction Power	[−0.001, 0.001]	0	0.5420
Mean Lateral Pole Force *	[−0.068, −0.019]	0.0116	**0**.**0045**
Mean Posterior Pole Force	[−0.025, 0.006]	0.0017	0.3659

The bold values correspond to *p* < 0.05.

* Added to the variable name when *p* < 0.05.

## 4 Discussion

This study investigated the effects of pole position and grip height on kinetic parameters and throwing performance in seated shot put across four F33–34 athletes. The principal findings were that pole position significantly affected seven of thirteen kinetic variables, while grip height and the pole position  ×  grip height interaction had no significant effects after FDR correction. Despite these substantial kinetic changes, throw distance was not significantly affected by any pole configuration, a pattern consistent with, but not directly demonstrative of, compensatory movement strategies (release kinematics and segmental sequencing were not analysed here and would be required to identify the specific mechanism by which performance was preserved). Of the seven kinetic variables influenced by pole position, only mean lateral pole force was significantly associated with within-athlete throw distance, identifying it as the sole pre-selected variable that was both altered by equipment configuration and significantly associated with within-athlete throw distance. Regarding the study hypotheses, the first hypothesis was supported: pole position significantly affected upper-body kinetic parameters consistently across multiple athletes. The second hypothesis was not supported: grip height had no significant effect on any kinetic variable after correction for multiple comparisons. The third hypothesis was partially supported: while the majority of kinetic changes induced by pole position did not translate into performance differences, mean lateral pole force was the notable exception, showing a significant within-athlete association with throw distance.

### 4.1 Pole position as the primary determinant of kinetic strategy

All seven significant kinetic effects were attributable to pole position, with no grip height main effects or interaction effects surviving FDR correction. The dominance of pole position over grip height is consistent with the biomechanical rationale that horizontal repositioning of the pole alters the moment arm between the athlete's centre of mass and the support point, thereby fundamentally changing the force-generation demands during the throwing motion. By contrast, the grip height increments used in this study (75 mm) were half the magnitude of the pole position increments (150 mm), and their effects may have been too subtle to detect given the sample size. This finding extends the single-athlete observations of O’Riordan et al. (3) and Holdback et al. (2) by demonstrating that pole position effects on trunk kinetics are consistent across multiple athletes within the F33–34 classification. Two non-mutually-exclusive explanations should be acknowledged for the null grip-height result: (i) the smaller grip-height perturbation (75 mm vs. 150 mm for pole position) may have produced biomechanical changes below the sensitivity threshold demonstrated above (Cohen's d ≥ ∼0.8 after FDR correction); and (ii) the limited sample (*n* = 4) constrains statistical power for any moderate effect. These explanations cannot be disentangled with the present data, and the null grip-height findings should therefore be interpreted as ‘no detectable effect under the present conditions’ rather than as evidence of a true absence of effect.

The pole force variables exhibited the largest effect sizes in the study. Mean posterior pole force increased substantially in the Far condition (d = 2.67 vs. Close; d = 2.33 vs. Normal), reflecting the greater posterior force required to maintain stability when the pole is positioned farther from the athlete. Among the trunk variables, axial rotation power showed the strongest response to pole position (q < 0.001), with the Far and Normal positions eliciting greater trunk rotation power than the Close position (d = 1.15 and d = 0.87, respectively). This aligns with the expectation that a more distant pole increases the rotational demands on the trunk during the throwing motion. In contrast, trunk flexion power was greatest in the Normal position relative to Far (d = 0.80), while trunk lateral flexion power was greatest in the Close position relative to Far (d = 0.69), suggesting that different pole positions preferentially load different trunk movement planes.

The throwing-arm kinetic responses further illustrate how pole position redistributes the mechanical demands of the throw. Shoulder horizontal adduction power was greater in the Normal and Close conditions compared to Far (d = 0.80 and d = 0.70), whereas shoulder abduction power was greater in the Normal and Far conditions compared to Close (d = 0.84 and d = 0.73). These opposing patterns suggest that pole position shifts the relative contributions of different shoulder actions, potentially reflecting changes in the orientation of the throwing arm relative to the trunk at the point of force application. It is noteworthy that grip height approached FDR-corrected significance for throwing-arm shoulder horizontal adduction power (q = 0.053), raising the possibility that grip height effects on specific upper-limb variables may emerge with greater statistical power.

### 4.2 Biomechanical compensation and the preservation of throw distance

Perhaps the most notable finding of this study was the disconnection between kinetic changes and throwing performance. Despite effect sizes as large as d = 2.67 for posterior pole force, pole configuration explained less than 1% of the variance in throw distance (marginal R^2^ = 0.005), with 92% of distance variance attributable to between-athlete differences (ICC = 0.92). This pattern is consistent with the concept of motor abundance (17), whereby the musculoskeletal system possesses sufficient degrees of freedom to achieve equivalent task outcomes through different coordination strategies. In the context of seated shot put, athletes appear capable of redistributing joint powers and pole forces across different configurations while maintaining comparable release parameters and thus similar throw distances.

This finding mirrors observations in earlier seated throwing research. O’Riordan et al. (3) reported that changing pole placement increased trunk rotational velocity but did not translate into an immediate performance improvement for a single athlete. Holdback et al. (2) similarly showed that altering grip height produced kinetic changes without immediate performance gains, though performance improved after several practice sessions as the athlete adapted to the new configuration. The present study extends these single-case observations to a group of four athletes and a broader set of kinetic variables, confirming that the compensatory phenomenon is robust across individuals within the F33–34 classification. An important practical implication follows: coaches can manipulate pole position to alter the mechanical loading on specific joints, for example, using a far position to increase trunk axial rotation demands, without risking acute performance decrements.

### 4.3 Mean lateral pole force as the performance-relevant variable

Among all kinetic variables, mean lateral pole force was unique in satisfying two criteria: it was significantly altered by pole position, and it was significantly associated with within-athlete throw distance (although the magnitude of that association was modest). The negative within-athlete association (beta = −4.3 cm per 1 N increase; mean within-athlete r = −0.292) indicates that a shift toward more laterally directed (more negative) pole force was associated with greater throw distance. The Far pole position produced the least laterally directed force (EMM = 3.05 N), while the Close and Normal positions allowed a more lateral force strategy, suggesting that closer pole placements may facilitate a lateral force application pattern that is more favourable for performance.

Biomechanically, lateral pole force may reflect the athlete's ability to generate a reaction force that assists medio-lateral stability or contributes to trunk rotation during the throwing motion. The very high ICC for this variable (0.976) indicates that the magnitude and direction of lateral pole forces are highly athlete-specific, representing an individual technique signature that remains stable across configurations. Pole position shifts the magnitude of this force within each athlete's characteristic pattern rather than fundamentally altering the pattern itself. This interpretation is supported by the small marginal R^2^ for the pole configuration model (0.006) relative to the conditional R^2^ (0.976), indicating that nearly all variance in lateral pole force is explained by athlete identity rather than by experimental condition. We emphasise that this within-athlete association, although statistically significant after FDR correction, explained only about 1.2% of within-athlete throw distance variance (*Δ*R^2^ = 0.012; mean within-athlete r = −0.292). The practical magnitude of the association is therefore small, and the result should be regarded as preliminary and hypothesis-generating rather than as a robust performance predictor.

It is worth noting that mean posterior pole force, despite exhibiting the largest effect sizes in the study (d = 2.33–2.67), showed no significant within-athlete association with throw distance (q = 0.366). This disconnection suggests that posterior pole force is a mechanical consequence of pole placement, where athletes must pull harder against a more distant pole to maintain equilibrium, rather than a direct contributor to throwing performance. The remaining five significant trunk and shoulder variables likewise showed no performance association (all q > 0.35), reinforcing the conclusion that the kinetic changes induced by pole position in these variables represent compensatory adjustments that do not translate into measurable distance differences.

### 4.4 Practical implications for coaching and equipment optimisation

Several practical implications emerge from these findings. First, pole position selection should be individualised based on athlete-specific force profiles. Given the highly athlete-specific nature of lateral pole force (ICC = 0.976) and its association with performance, instrumented pole force assessment may be a useful exploratory tool for characterising athlete-specific force profiles, although the modest predictive magnitude (*Δ*R^2^ = 0.012) indicates that any such individualisation should be validated longitudinally before being adopted as routine practice. Second, the absence of significant grip height effects simplifies the equipment optimisation process for coaches, as pole position appears to be the more impactful variable to prioritise. Third, the disconnection between kinetic changes and throw distance suggests that pole position may be a candidate variable for training-load modulation, that should be evaluated in dedicated longitudinal studies before being adopted in practice. For example, a far pole position could be employed to increase trunk axial rotation demands during training sessions, potentially strengthening the relevant musculature without acute performance decrement in the present single-session protocol; whether this holds with prolonged use, transfer, or in competition contexts is not tested here.

For F33–34 athletes specifically, the variance structure of the data carries additional implications. The high ICCs observed for pole forces and several shoulder variables indicate that these kinetic profiles are individual “signatures” shaped by each athlete's unique impairment characteristics and learned movement strategies. This is consistent with the known heterogeneity within Paralympic classifications (10), where athletes with coordination impairments present highly individualised functional profiles. Coaching recommendations should therefore be tailored to the individual rather than applied uniformly across athletes within the same class.

### 4.5 Limitations

Several limitations should be acknowledged. The sample comprised only four athletes, which, while typical of the challenges in elite Paralympic research where athlete pools are inherently small, limits statistical power for detecting smaller effects and precludes the use of random-slope models that could capture athlete-specific responses to pole configuration. The absence of significant grip height and interaction effects may reflect insufficient power rather than true null effects. The random-intercept-only model structure means that the estimated fixed effects represent average responses across athletes and may not accurately characterise the response of any individual athlete. Future studies with larger cohorts, maybe with international collaboration, would enable random-slope models to address this limitation. The cohort included an equal sex distribution (two male, two female), but with *n* = 2 per sex the design cannot inform any sex-specific conclusion about pole-configuration effects on kinetics; sex-stratified individual-level kinetic profiles were not reported in the figures because of re-identification risk in the small F33–34 classification pool. Block order of the three pole-position conditions was not fully counterbalanced: the Normal condition was tested first for every athlete (to establish the reference frame from which the ±150 mm Close and Far shifts were measured and to provide opening throws for the end-of-session fatigue check), and only the Close and Far blocks were alternated between athletes. This design therefore does not control for order or warm-up effects on the Normal condition itself, although the absence of a significant decrement between the opening and closing Normal blocks (d = 0.24) is consistent with any such effects being small. Finally, with 2–3 trials per cell the within-condition, within-athlete reliability estimates carry wide uncertainty and should be regarded as descriptive only. Because the model included a random intercept only, it tests the group-average effect of pole configuration; it does not test, and cannot evidence, whether the direction or magnitude of that effect is consistent at the individual athlete level. Apparent cross-athlete consistency in Figures 2 and 3 should therefore be regarded as descriptive only.

Regarding model diagnostics, nine of thirteen models exhibited non-normal residuals (Shapiro–Wilk *p* < 0.05). However, the two key variables underpinning the central finding, mean lateral pole force (*p* = 0.076) and mean posterior pole force (*p* = 0.582), had well-behaved residuals. Furthermore, all seven significant pole position effects were validated by permutation tests and survived log-transform sensitivity analyses, providing confidence in the robustness of the results. Only one model showed evidence of heteroscedasticity (trunk axial rotation power, Levene's *p* = 0.040), and no models had singular fits or influential observations driving the conclusions (maximum Cook's D = 0.105).

The within-athlete performance associations, while embedded within an experimental design, are observational in nature and should be interpreted as associations rather than causal relationships. Additionally, this was a cross-sectional study in which athletes were tested in a single session; the acute kinetic responses observed here may not reflect chronic adaptations that would develop over prolonged training with a new pole configuration, as suggested by the longitudinal improvements reported in Holdback et al. (2). Finally, the study was limited to F33–34 athletes with coordination impairments. Other seated throwing classes, particularly F51–57 athletes with muscle weakness or limb difference, may respond differently to pole configuration changes due to their distinct functional limitations.

### 4.6 Future directions

Future research should aim to recruit larger cohorts of seated throwers to improve statistical power for detecting grip height effects and to enable random-slope models that can characterise individual athlete responses. Longitudinal studies examining how athletes adapt to new pole configurations over training blocks would clarify whether the acute compensatory strategies observed here evolve into stable, performance-enhancing movement patterns with practice. Extending this investigation to other seated throwing classifications (F31–32, F54–57) would determine whether the dominance of pole position over grip height is consistent across different impairment types. Furthermore, covering other athlete-to-pole set-up, such as pole position change in the medio-lateral plane and pole angle change relative to the ground, would help unravel the best set-up for maximum performance. Complementary kinematic analyses would help elucidate the movement coordination strategies that underpin the kinetic changes documented here, providing a more complete picture of how athletes reorganise their technique in response to equipment changes. Finally, investigating whether individual athletes utilise optimal lateral pole force profiles that can be identified and trained represents a promising avenue for personalised equipment optimisation in Paralympic seated throwing.

### 4.7 Conclusion

This study demonstrates that pole position was the more influential of the two equipment variables examined in this dataset, influencing the kinetic strategy of seated shot put in this cohort of F33–34 athletes, while grip height and their interaction had no detectable effects. Athletes maintained comparable throw distances across all nine pole configurations despite substantial changes in trunk, shoulder, and pole force kinetics, indicating an apparent compensatory capacity within the throwing system that warrants direct testing with release-kinematic and coordination measures. Mean lateral pole force emerged as the sole kinetic variable that was both sensitive to pole position and significantly associated with within-athlete throw distance, with a more laterally directed force associated with greater distance. These findings provide a biomechanical basis for individualised pole position selection in Paralympic seated throwing and suggest that pole configuration can be leveraged as a targeted training tool to modulate joint loading without acute performance decrement in this single-session protocol. More broadly, the kinetic ’signature’ visible across F33–34 athletes, and the dissociation between large kinetic changes and small distance changes, suggests that within-class equipment standardisation should not be assumed to be optimal at the athlete level, and that biomechanical individualisation is a coherent next step for Paralympic seated throwing practice.

## Figures and Tables

**Figure B1:**
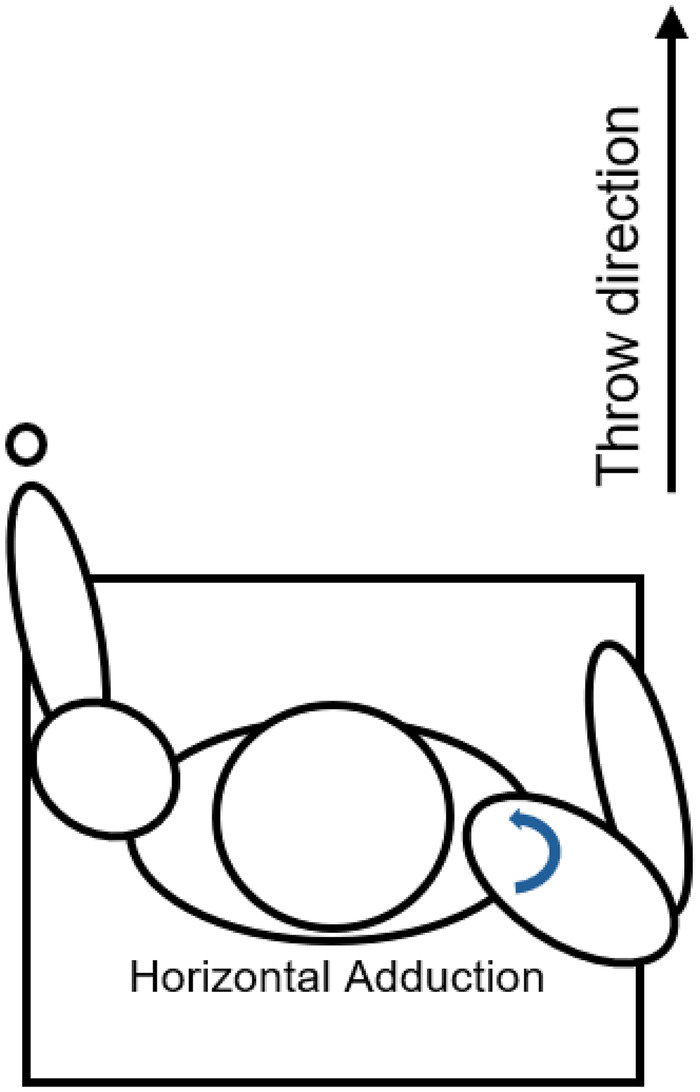
Horizontal Adduction of the Shoulder defined through the vertical axis of the trunk segment.

**Figure C1:**
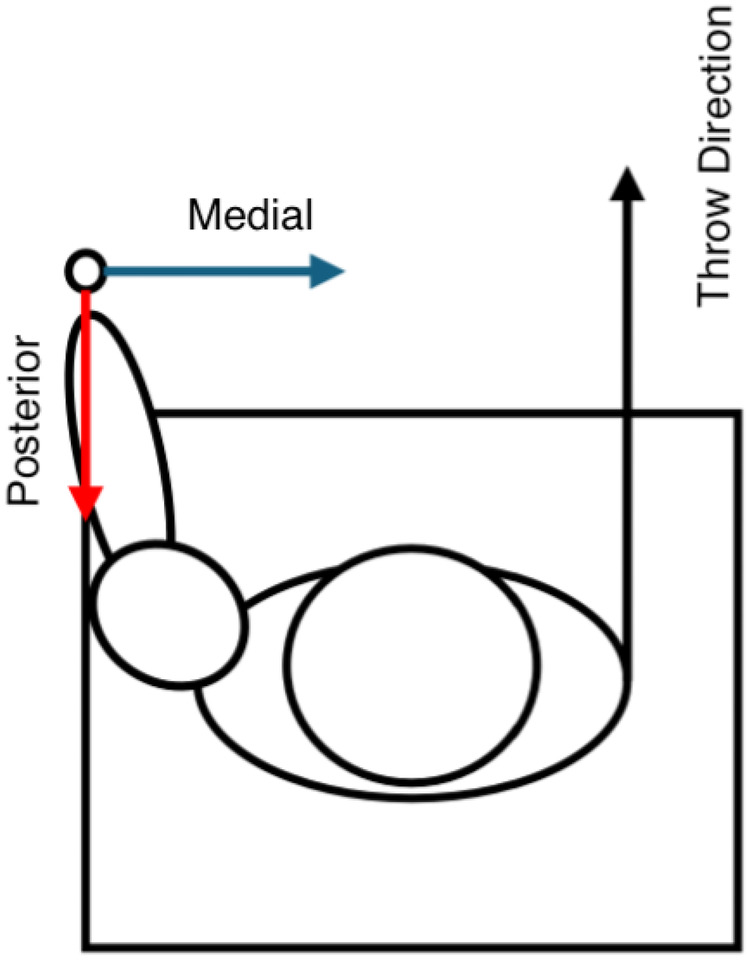
Definition of Posterior, and Lateral (opposite to Medial) Pole Forces.

## Data Availability

The datasets presented in this article are not readily available because of confidentiality agreements with the national sporting organization. Requests to access the datasets should be directed to Rony Ibrahim, rony.ibrahim@qu.edu.qa.

## Appendix A

Table A.1: Defining body segments and their anatomical landmarks used in the present study.

**Table:**

Body segments	Corresponding anatomical landmarks
Pelvis	Right and left antero-superior iliac spines; midpoint between the postero-superior iliac spines; navel
Abdomen	Navel; 12th thoracic (T12); xiphoid process
Thorax	Xiphoid process; 6th thoracic (T6); suprasternal; 7th cervical (C7)
Head	7th cervical (C7); right and left tragion; head vertex
Upper arm	Acromion process; lateral and medial humeral epicondyles
Forearm	Lateral and medial humeral epicondyles; radial and ulnar styloids

## Appendix B

Horizontal Adduction of the shoulder joint was defined to represent the motion of the humerus across the midline of the trunk in a simpler way than would be possible with pure anatomical rotations of the shoulder joint (flexion, adduction, rotation). Horizontal adduction is especially applicable in the shot put motion when compared to rotation.
